# The combination of UHPLC-HRMS and molecular networking improving discovery efficiency of chemical components in Chinese Classical Formula

**DOI:** 10.1186/s13020-021-00459-6

**Published:** 2021-07-02

**Authors:** Xiaoxia Xue, Qishu Jiao, Runa Jin, Xueyuan Wang, Pengyue Li, Shougang Shi, Zhengjun Huang, Yuntao Dai, Shilin Chen

**Affiliations:** 1 Institute of Chinese Materia Medica, China Academy of Chinese Medical Sciences, Beijing, 100700 China; 2 Shanxi University of Chinese Medicine, Jinzhong, 030619 Shanxi China; 3 Modern Research Center for Traditional Chinese Medicine, Shanxi University, Taiyuan, 030006 Shanxi China; 4 Sunflower Pharmaceutical Group (Xiangyang) Longzhong Co. Ltd, Xiangyang, 441003 Hubei China

**Keywords:** Chinese Classical Formula, Chemical component identification, Erdong decoction, UHPLC-LTQ-Orbitrap-MS/MS, Molecular networking, Steroidal saponins, Triterpenoid saponins, Flavonoid *C*-glycosides

## Abstract

**Background:**

It is essential to identify the chemical components for the quality control methods establishment of Chinese Classical Formula (CCF). However, CCF are complex mixture of several herbal medicines with huge number of different compounds and they are not equal to the combination of chemical components from each herb due to particular formula ratio and preparation techniques. Therefore, it is time-consuming to identify compounds in a CCF by analyzing the LC–MS/MS data one by one, especially for unknown components.

**Methods:**

An ultra-high pressure liquid chromatography-linear ion trap-orbitrap high resolution mass spectrometry (UHPLC-LTQ-Orbitrap-MS/MS) approach was developed to comprehensively profile and characterize multi-components in CCF with Erdong decoction composed of eight herbal medicines as an example. Then the MS data of Erdong decoction was analyzed by MS/MS-based molecular networking and these compounds with similar structures were connected to each other into a cluster in the network map. Then the unknown compounds connected to known compounds in a cluster of the network map were identified due to their similar structures.

**Results:**

Based on the clusters of the molecular networking, 113 compounds were rapidly tentative identification from Erdong decoction for the first time in the negative mode, which including steroidal saponins, triterpenoid saponins, flavonoid *O*-glycosides and flavonoid *C*-glycosides. In addition, 10 alkaloids were tentatively identified in the positive mode from Nelumbinis folium by comparison with literatures.

**Conclusion:**

MS/MS-based molecular networking technique is very useful for the rapid identification of components in CCF. In Erdong decoction, this method was very suitable for the identification of major steroidal saponins, triterpenoid saponins, and flavonoid *C*-glycosides.

**Supplementary Information:**

The online version contains supplementary material available at 10.1186/s13020-021-00459-6.

## Background

The Chinese Classical Formula (CCF) are the essences of thousands of years of practical experience in the clinical application of traditional Chinese medicines (TCM). It is important and preferred direction of traditional Chinese medicine (TCM) to develop CCF into modern preparations to meet the needs of convenience. The chemical components analysis is of great significance for the study of pharmacologically active components and the establishment of quality control methods of CCF. The main chemical components of CCF are extremely complex and they are not equal to the combination of chemical components of each herb due to different formula proportions and preparation techniques. Therefore, how to quickly identify the main chemical components of a TCM formula is an important step for the modernization development of CCF.

Identification of chemical components of TCM formula have been facilitated by modern analytical techniques. In particularly, high-resolution mass spectrometry (HRMS) plays a critical role in characterizing structures of chemical compounds by providing precise molecular weight as well as fragmental structures with the advantages of high sensitivity and throughput in detecting versatile molecules [1]. Conventionally, liquid chromatography mass spectrometry (LC–MS) is one of the most widely used approaches to the preliminary characterization of chemical components of TCM formula extract. Nevertheless, it is time-consuming and difficult to analyze the MS data of a TCM formula due to its complex components, especially for unknown components.

Recently, the combination of LC-HRMS and molecular networking has facilitated the MS data analysis. Molecular networking (MN) is outstanding to dispose of complicated MS data. It is capable of gathering the molecules with similar structures together based on the similarity of their MS/MS fragments. Compounds that share similar MS/MS fragmentation patterns or molecular classes are likely to group together in MN. This improves the possibility of identification of unidentified nodes, if their spectra or the spectra of surrounding nodes are known by references [2–4]. Thus, the combination of LC-HRMS and molecular networking immensely enhances the efficiency and drastically reduces the time on data processing. In the last few decades, molecular networking was introduced in drug development and metabolomics, particularly for natural products containing hundreds of components.

As one example from the "Catalogue of Ancient Chinese Classic formula (First Batch)", Erdong decotion was record in *yixuexinwu* and used in nourishing Yin and quenching thirst. In modern clinical practice, Erdong decoction and its modified prescriptions have been mainly used to treat type 2 diabetes and its complications [5, 6]. It was composed of eight herbs including Asparagi Radix (the root of *Asparagus cochinchinensis* (Lour.)Merr.), Ophiopogonis Radix (the root of *Ophiopogon japonicus*.), Trichosanthis Radix (the root of *Trichosanthes kirilowii* Maxim.), Scutellariae Radix (the root of *Scutellaria baicalensis* Georgi.), Anemarrhenae Rhizoma (the rhizome of *Anemarrhena asphodeloides* Bunge.), Glycyrrhizae Radix Et Rhizoma (the root et rhizome of *Glycyrrhiza uralensis* Fisch.), Ginseng Radix Et Rhizoma (the root et rhizome of *Panax ginseng* C. A. Mey.) and Nelumbinis Folium (the leaf of *Nelumbo nucifera* Gaertn.). However, hitherto there is no report on systematic characterization of chemical components of Erdong decoction and its quality control methods.

**Fig. 1:**
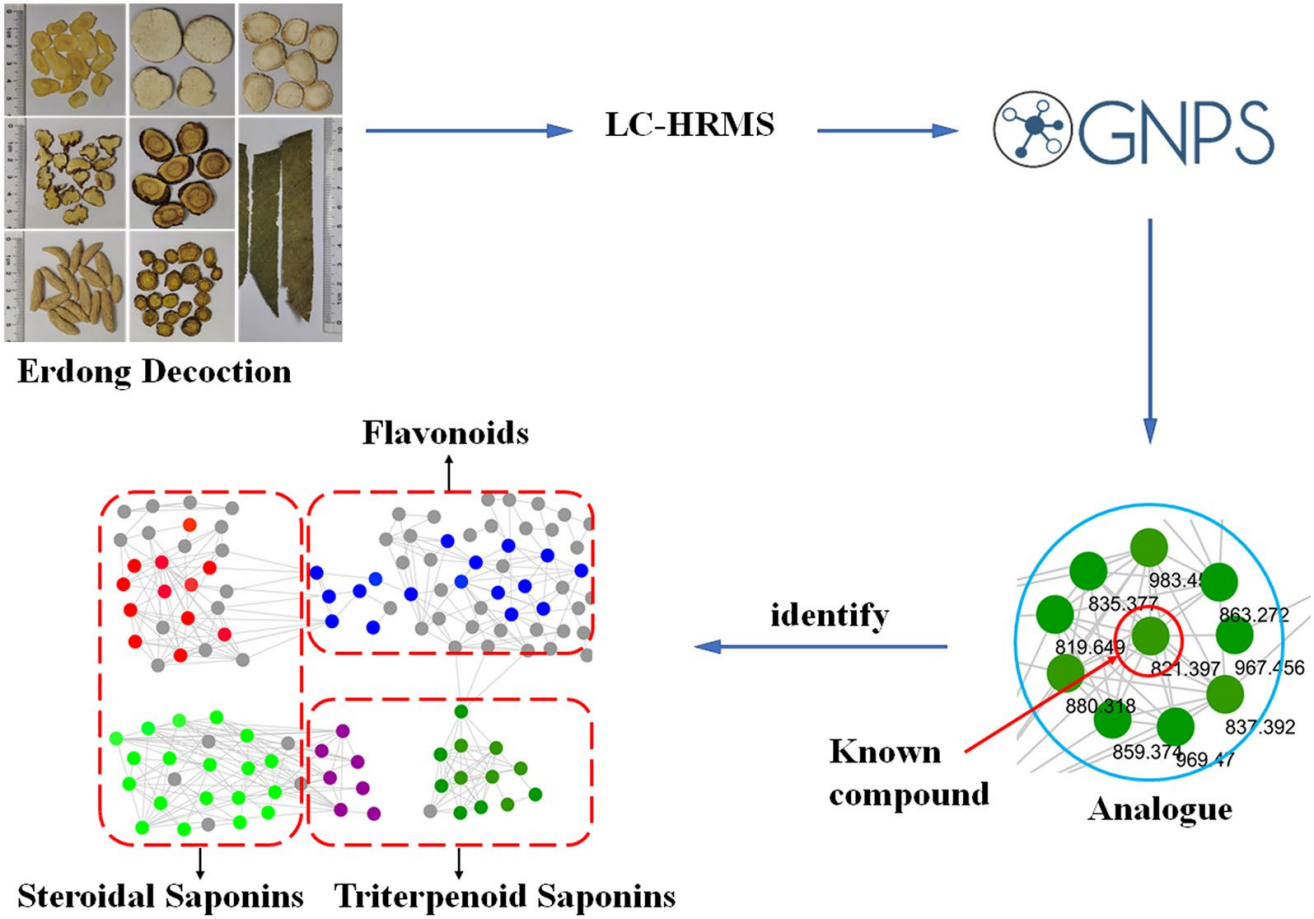
A general workflow for a strategy identifying compounds rapidly of Erdong decoction

In this study, the combination of LC-HRMS and molecular networking was applied to rapidly identify compounds in Erdong decoction as a case study to demonstrate the application of the combined techniques in TCM formula. An ultra-high pressure liquid chromatography-linear ion trap-orbitrap high resolution mass spectrometry (UHPLC-LTQ-Orbitrap-MS/MS) approach was developed to comprehensively profile and characterize multi-components in Erdong decoction. Then the MS data of Erdong decoction was analyzed by MS/MS-based molecular networking (Fig. 1). The results show that the combination of LC-HRMS and molecular networking greatly improves the efficiency of chemical components identification in CCF composed of many herbs.

## Materials and methods

### Materials and reagents

*Asparagus cochinchinensis* was purchased from Guizhou Province in July 2018. *O. japonicus* was purchased from Santai, Sichuan Province in July 2018. *T. kirilowii* was purchased from Feicheng, Shandong Province in July 2018. *S. baicalensis* was purchased from Lingchuan, Shanxi Province in July 2018. *A. asphodeloides* was purchased from Wanrong, Shanxi Province in July 2018. *G. uralensis* was purchased from Beitun Town, Xinjiang Province in July 2018. *P. ginseng* was purchased from Fushong, Jilin Province in July 2018. *N. nucifera* was purchased from Nanchang, Jiangxi Province in September 2018. Reference compounds, neomangiferin, oroxylin A-7-*O*-*β*-D-glucuronide and glycyrrhizin acid were purchased from Beijing Century Aoko Biotechnology Co. Ltd. (Beijing, China), mangiferin, baicalin and wogonoside were purchased from National Institutes for Food and Drug Control (Beijing, China), and quercetin-3-*O*-glucuronide and hyperoside were purchased from Chengdu Cloma Biological Technology Co. Ltd. (Sichuan, China). HPLC-grade acetonitrile and LC–MS-grade formic acid were purchased from Fisher Scientific (USA).

### Sample preparation

The solutions of neomangiferin, mangiferin, hyperoside, quercetin-3-*O*-glucuronide, baicalin, oroxylin A-7-*O*-*β*-D-glucuronide, wogonoside and glycyrrhizic acid were prepared in methanol at appropriate concentrations. A mixture of 8 different slices consisting of 33.6 g of dried *O. japonicus* radixs, 22.5 g of dried *A. cochinchinensis* radixs, 11.1 g of dried *T. kirilowii* radixs, 11.1 g of dried *S. baicalensis* radixs, 11.1 g of dried *A. asphodeloides* naerhizomas, 11.1 g of dried *N. nucifera* foliums, 5.7 g of dried *G. uralensis* radix et rhizoma, and 5.7 g of dried *P. ginseng* radix et rhizome were subjected to decoction twice with 10-times amount of distilled water for 40 min and 6-times distilled water for 30 min, respectively. The extraction temperature is around 96–100 ℃, at which the decocting liquid keep boiling. All extraction solutions were concentrated to 560 mL at 60 ℃. One hundred microlitre of concentrated solution was dissolved in 900 μL of 10% acetonitrile and centrifuged at 13,000 r·min^−1^ for 5 min, then the supernatant solution was filtered through a 0.22 μm membrane filter prior to injection into the chromatographic system.

### Data acquisition and molecular networking analysis

HPLC analysis was performed on DIONEX Ultimate 3000 UHPLC system (USA) with photodiode array (PDA) detector. Samples were separated on an Acquity UPLC HSS T3 column (100 × 2.1 mm i.d., 1.8 μm) at 40 ℃. The mobile phase consisted of acetonitrile (A) and water containing 0.1% formic acid (B). A gradient program was adopted as follows: 0–3 min, 10–13% A; 3–6 min, 13–14% A; 6–9 min, 14–17% A; 9–11 min, 17–25% A; 11–18 min, 25–30% A; 18–19 min, 30–48% A; 19–22 min, 48–48% A, with a flow rate of 0.4 mL/min. The PDA detector scanned at 254 nm.

The LTQ-Orbitrap XL mass spectrometer was purchased from Thermo Scientific equipped with electrospray ionization (EIS) and Xcalibur 2.1 workstation. The analysis was performed in both negative and positive mode with a mass range of m/z 100–1400. High-purity nitrogen (N_2_) was used as auxiliary gas (10 arb) and sheath gas (40 arb). The other parameters were as follows: capillary temperature, 350℃; capillary voltage, 3.3 kV (in the positive mode), 3.0 kV (in the negative mode).

The MS data of the targeted fraction was converted from the raw format to the mzXML format using the Proteo-Wizard 3.0.20014. Then, the mzXML file was uploaded by the suggested software of WinSCP (https://winscp.net/eng/download.php) to the GNPS platform (https://gnps.ucsd.edu). The resulting analysis and parameters for the network can be accessed via links http://gnps.ucsd.edu/ProteoSAFe/status.jsp?task=4e68c1650ff24c9091a7a021d52531e0 (in the negative mode) and http://gnps.ucsd.edu/ProteoSAFe/status.jsp?task=bcd0018bf90d44c09353515f1ed7bdca (in the positive mode). The following settings were used for generation of the network: minimum pairs cos 0.6; parent mass tolerance, 2 Da; MS/MS fragment ion tolerance, 0.5 Da; network top, 10; minimum matched peaks, 5. The molecular networking data were analyzed and visualized using Cytoscape (ver. 3.7.2).

## Results

### Study on molecular networking of mass spectrometry of Erdong decoction

**Fig. 2:**
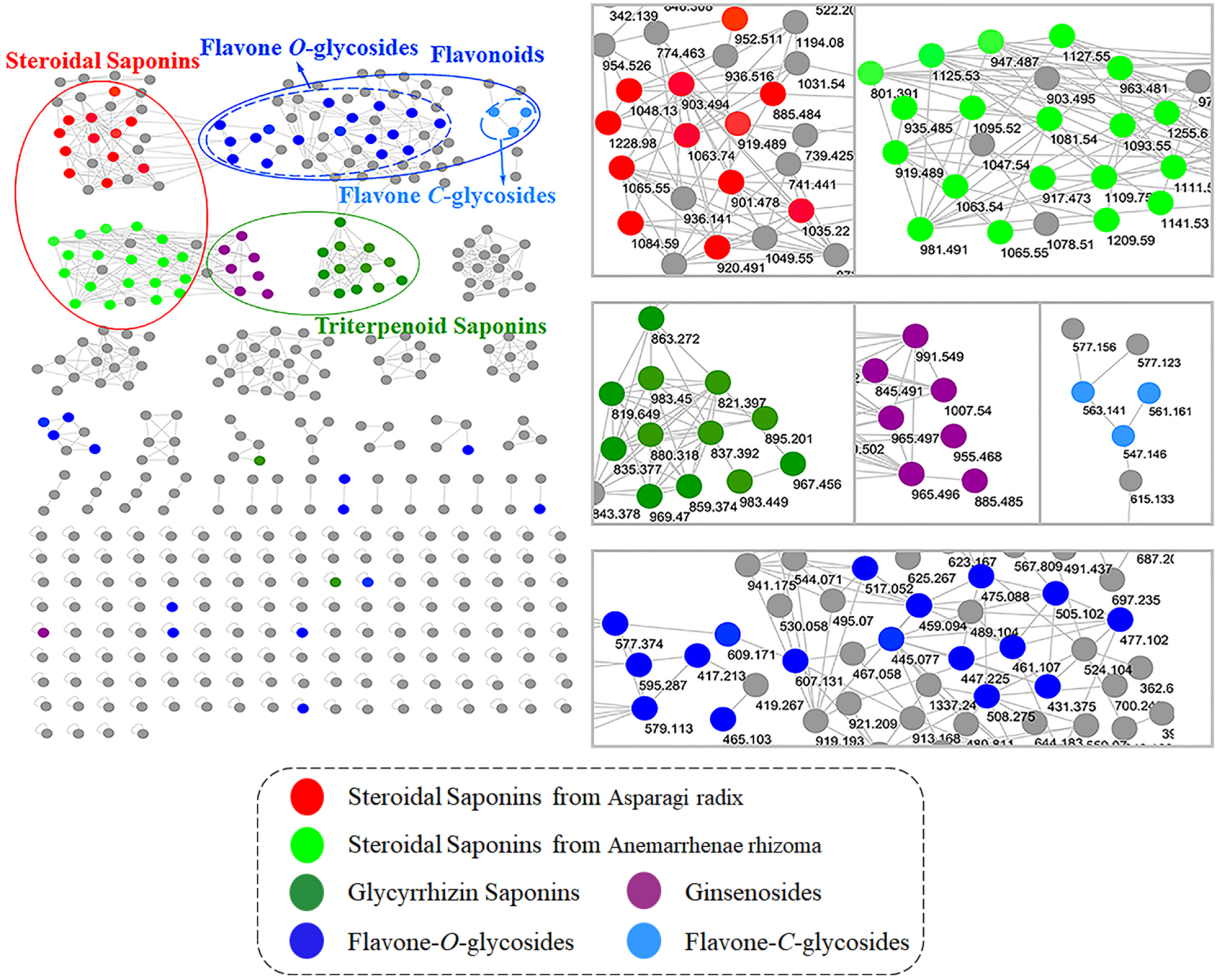
MS/MS molecular networking of Erdong decoction in the negative mode

**Fig. 3:**
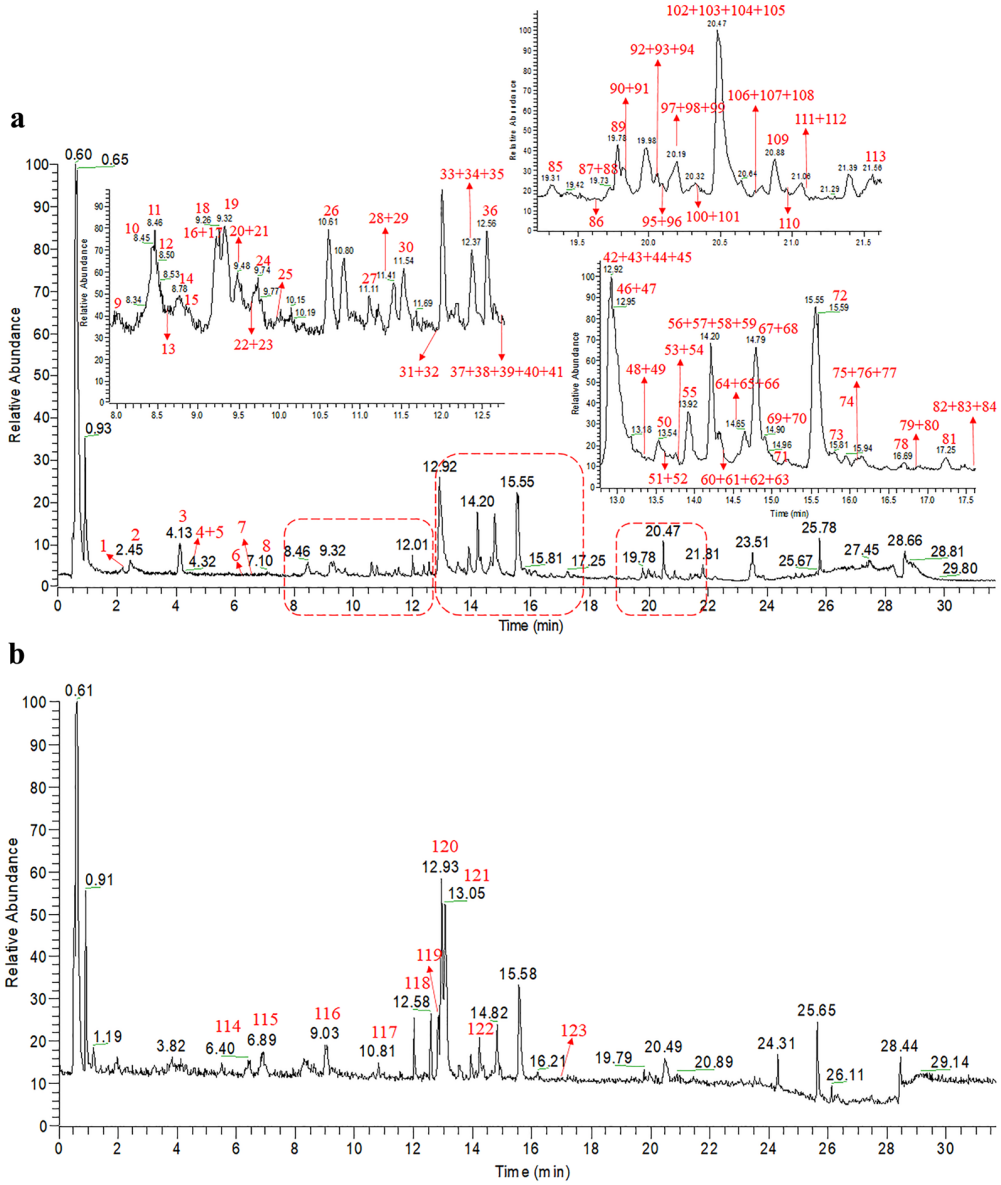
TIC of Erdong decoction in the negative mode (**a**) and positive mode (**b**)

All the full-MS and MS/MS spectra were obtained in high-resolution FT-MS for robust identification. In order to quickly identify the main chemical components in Erdong decoction, LC–MS/MS based molecular networking was applied. The MS data was processed through GNPS online workflow and visualized by MS/MS molecular networking. Their spectral similarities were evaluated through cosine calculation (cos θ), the larger the cos θ value, the higher the similarity of the MS/MS fragments [7]. The results showed that the cluster of molecular networking in the negative mode (Fig. 2) was more obvious than that of the positive mode (Additional file 1: Figure S1). The MS data of steroids, triterpenes, and flavonoids in the LC–MS/MS molecular networking of Erdong decoction were split into different groups. Herein, a total of 430 nodes was incorporated into the MS/MS molecular networking of Erdong decoction in the negative mode, rendering 30 molecular clusters and 164 unconnected nodes (Fig. 2). Based on the clusters in the molecular networking, 113 compounds were rapidly tentative identification from Erdong decoction for the first time in the negative mode, which including steroidal saponins, triterpenoid saponins, flavonoid *O*-glycosides and flavonoid *C*-glycosides. The typical total ion chromatograms (TIC) of Erdong decoction in the positive mode and the negative mode are presented in Fig. 3. Details of the characterization of these compounds were further elaborated.

### Rapid identification of steroidal saponins

**Fig. 4:**
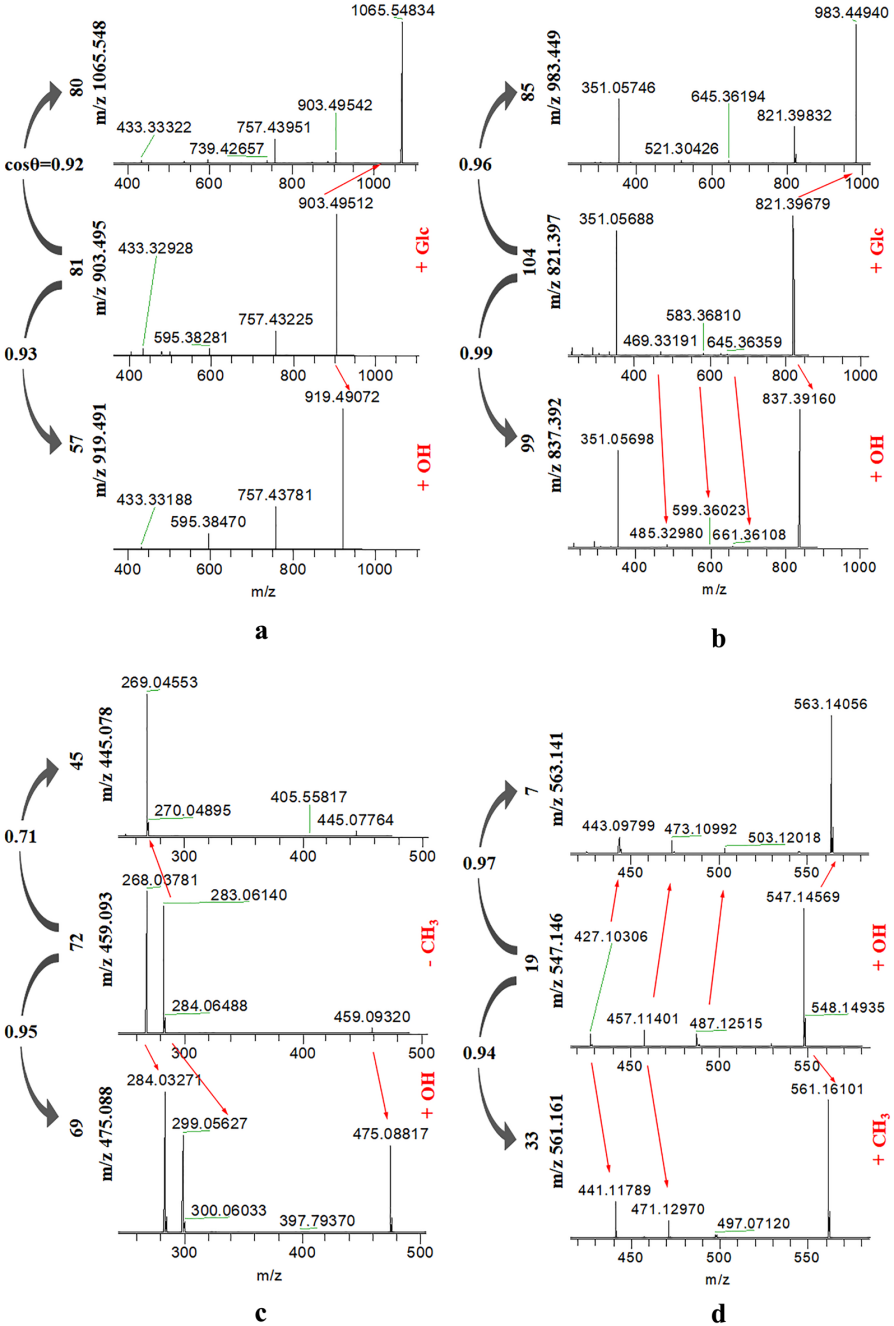
MS/MS spectra of **a** steroidal saponins, **b** triterpenoid saponins, **c** flavonoid *O*-glycosides and **d** flavonoid *C*-glycosides

Previous studies had reported that steroidal saponin was one of the main compounds of Asparagi radix [8]. Taking aspacochioside A at *m/z* 903.495 as an example, its MS/MS spectrum showed three characteristic fragments of *m/z* 757.432, *m/z* 595.383, and *m/z* 433.330, which in turn lost rhamnosyl, glucosyl and glucosyl, the fragment of *m/z* 433.330 corresponding to the aglycone of aspacochioside A (Additional file 1: Figure S2). The fragmentation scheme of aspacochioside A was further elaborated in Additional file 1: Figure S2. In comparison to aspacochioside A, its adjacent node of *m/z* 919.491 gave a MS/MS spectrum showing identical aglycone and three identical characteristic fragments, with different [M−H]^−^ ion (Fig. 4a). The node of *m/z* 919.491 was preliminarily deduced as aspacochioside A analogue with one more hydroxyl group to the rhamnose of aspacochioside A, finally annotated as 3-*O*-*β*-d-glucopyranosyl (1 → 2)-*β*-d-glucopyranosyl-26-*O*-*β*-d-glucopyranosyl-(25*S*)-5*β*-furostane-3*β*,22*α*,26-triol according literature [8]. According to the clusters, the structures of these compounds could be rapidly identified. Sixteen steroidal saponins were tentatively identified from Asparagi radix and 14 steroidal saponins were tentatively identified from Anemarrhenae rhizoma by comparison with reported literatures [8–10] (Table 1), and they were annotated in red and light green in Fig. 2, respectively.

**Table 1:** Tentative identification of the chemical constituents of Erdong decoction by UHPLC- MS in the negative mode

Peak no.	T_R_ (min)	Formula	Adduct ion	Experimental mass m/z	Theoretical mass m/z	Mass error (ppm)	Fragment ions	Identification	Source
1	2.21	C_25_H_28_O_16_	[M−H]^−^	583.1306	583.1294	2.039	493.0986, 463.0880, 301.0355, 259.0246, 244.0359	Neomangiferin	A
2	2.54	C_21_H_22_O_12_	[M−H]^−^	465.1032	465.1028	0.984	303.0524, 285.0406, 177.0184	Spiraeoside	S
3	4.13	C_19_H_18_O_11_	[M−H]^−^	421.0778	421.0765	2.974	331.0460, 301.0355, 285.0410, 271.0248, 259.0247	Mangiferin	A
4	4.33	C_21_H_21_O_11_	[M−H]^−^	449.1087	449.1078	1.987	287.0553, 259.0620, 171.3426, 151.5096, 125.0229	Taxifolin 7-rhamnoside	N
5	4.49	C_19_H_18_O_11_	[M−H]^−^	421.0778	421.0765	3.045	331.0461, 301.0355, 285.0404, 258.0169	Isomangiferin	A
6	6.30	C_27_H_32_O_14_	[M−H]^−^	579.1721	579.1708	2.224	255.0662	Liquiritigenin 7,4'-di-*O*-glucopyranoside	G
7	6.65	C_26_H_28_O_14_	[M−H]^−^	563.1406	563.1395	1.826	503.1202, 473.1099, 443.0980, 383.0772, 353.0668, 203.0360	Apigenin 6-*C*-glucoside-8-*C*-arabinoside	S
8	7.12	C_26_H_28_O_16_	[M−H]^−^	595.1307	595.1294	2.317	300.0277, 271.0249, 243.0286, 178.9976	Quercetin-3-*O*-sambubioside	N
9	8.01	C_21_H_22_O_9_	[M−H]^−^	417.1194	417.1180	3.288	255.0661, 153.0180, 135.0074, 119.0487	Neoliquiritin	G
10	8.43	C_27_H_30_O_14_	[M−H]^−^	577.9597	577.9599	− 0.502	541.9865, 506.0078, 479.0923, 255.0665	5-Hydroxy-2-(4-hydroxyphenyl)-4-oxo-4H-chromen-7-yl 2-*O*-(6-deoxy-*α*-l-mannopyranosyl)-β-d-glucopyranoside	G
11	8.46	C_21_H_22_O_9_	[M−H]^−^	417.1191	417.1180	2.640	255.0661, 153.0180, 135.0074, 119.0487	Liquiritin	G
12	8.50	C_26_H_14_O_12_	[M−H]^−^	517.0400	517.0402	− 0.217	471.0382, 255.0655, 153.0182, 135.0076, 119.0488	1,1',3,4',5,6',8,8'-Octahydroxy-9H,9'H-2,2'-bixanthene-9,9'-dione	G
13	8.67	C_26_H_28_O_13_	[M−H]^−^	547.1458	547.1446	2.180	487.1227, 457.1145, 427.1230, 367.0822, 337.0720	Isomer of chrysin 6-*C*-arabinoside-8-*C*-glucoside	S
14	8.78	C_26_H_30_O_13_	[M−H]^−^	549.1614	549.1603	2.135	417.1192, 255.0661, 153.0180, 135.0074, 119.0486	Liquiritin apioside	G
15	8.86	C_32_H_40_O_18_	[M−H]^−^	711.2114	711.2131	− 2.335	549.1617, 255.0660, 153.0176, 135.0073, 119.0487	Glucoliquirtin asioside	G
16	9.22	C_21_H_20_O_12_	[M−H]^−^	463.0884	463.0871	2.694	301.0346, 300.0276, 272.0300, 271.0249, 255.0298, 178.9979, 151.0024	Hyperoside	N
17	9.23	C_27_H_30_O_16_	[M−H]^−^	609.1450	609.1450	0.015	300.0277, 271.0251, 255.0297, 178.9977	Rutin	N
18	9.26	C_21_H_20_O_10_	[M−H]^−^	431.0972	431.0973	− 0.100	341.0667, 311.0565, 283.0613, 269.0455	Baicalein 7-*O*-*β*-d-glucoside	S
19	9.33	C_26_H_28_O_13_	[M−H]^−^	547.1457	547.1446	1.961	487.1252, 457.1140, 427.1031, 367.0822, 337.0718	Chrysin 6-*C*-arabinoside-8-*C*-glucoside	S
20	9.42	C_26_H_30_O_14_	[M−H]^−^	565.1558	565.1552	1.023	438.8078, 295.0642, 271.0612	Hydroxyliquiritin apioside	G
21	9.48	C_21_H_18_O_13_	[M−H]^−^	477.0676	477.0664	2.585	302.0389, 301.0354, 283.0245, 255.0300, 227.0338, 178.9976, 151.0024	Quercetin-3-*O*-glucuronide	N
22	9.59	C_26_H_28_O_15_	[M−H]^−^	579.1363	579.1344	3.183	284.0328, 255.0293, 227.0346, 151.0025	Leucoside	N
23	9.68	C_23_H_24_O_13_	[M−H]^−^	507.1151	507.1133	3.595	345.0613, 330.0382, 315.0154	Viscidulin III-6'-*O*-*β*-d-glucopyranoside	S
24	9.74	C_21_H_20_O_12_	[M−H]^−^	463.0884	463.0871	2.694	300.0276, 271.0250, 255.0296, 178.9976, 151.0024	Isoquercitrin	N
25	9.86	C_21_H_18_O_12_	[M−H]^−^	461.0716	461.0715	0.234	285.0407, 267.0296, 175.0238	Scutellarin	S
26	10.61	C_26_H_28_O_13_	[M−H]^−^	547.1457	547.1446	1.961	457.1138, 427.1029, 367.0823, 337.0719	Chrysin 6-*C*-glucoside-8-*C*-arabinoside	S
27	11.11	C_26_H_28_O_13_	[M−H]^−^	547.1458	547.1446	2.070	457.1140, 427.1028, 367.0822, 337.0720	Isomer of chrysin 6-*C*-arabinoside-8-*C*-glucoside	S
28	11.26	C_27_H_28_O_16_	[M−H]^−^	607.1306	607.1294	2.074	431.0992, 269.0456	Trihydroxyflavone-glycoside glucuronide	S
29	11.35	C_27_H_28_O_16_	[M−H]^−^	607.1303	607.1294	1.563	445.0771, 431.0983, 269.0455	Trihydroxyflavone-glycoside glucuronide	S
30	11.59	C_21_H_20_O_9_	[M−H]^−^	415.1035	415.1024	2.629	295.0613, 267.0663, 251.0709, 223.0758	Chrysin 8-*C*-*β*-glucoside	S
31	11.82	C_23_H_24_O_10_	[M−H]^−^	459.1299	459.1286	2.781	255.0661, 153.0181, 135.0073, 119.0487	6'-Acetyliquiritin	G
32	11.91	C_23_H_24_O_13_	[M−H]^−^	507.1144	507.1133	2.037	344.0537, 329.0306, 316.0585	Viscidulin III-2'-*O*-*β*-d-glucopyranoside	S
33	12.35	C_27_H_30_O_13_	[M−H]^−^	561.1610	561.1603	1.324	471.1297, 441.1179, 281.0830	5-Hydroxy-7-methoxyflavone 6-*C*-arabinoside-8-*C*-glucoside or 7-hydroxy-5-methoxyflavone 6-*C*-arabinoside-8-*C*-glucoside	S
34	12.36	C_22_H_20_O_12_	[M−H]^−^	475.0880	475.0871	1.847	299.0563, 284.0327	Isomer of hydroxyl oroxylin A 7-*O*-glucuronide or hydroxyl wogonoside	S
35	12.37	C_45_H_76_O_20_	[M−H]^−^	935.4862	935.4846	1.699	773.4357, 611.3790, 449.3284	Timosaponin E	A
36	12.59	C_51_H_84_O_25_	[M−H]^−^	1095.5241	1095.5218	2.059	933.4723, 771.4182, 404.0874	(2*α*,3*β*,5*α*,6*β*,25*R*)-2,6-Dihydroxyspirostan-3-yl-*β*-d-glucopyranosyl-(1 → 2)-[*β*-d-glucopyranosyl-(1 → 3)]-*β*-d-glucopyranosyl-(1 → 4)-*β*-d-galactopyranosid	A
37	12.65	C_32_H_26_O_11_	[M−H]^−^	585.1365	585.1391	− 4.440	549.1618, 539.2637, 417.1174, 297.0774, 255.0662	{3-(4-Hydroxy-3-methoxyphenyl)-6-[(2*R*,3*R*)-3,5,7-trihydroxy-4-oxo-3,4-dihydro-2H-chromen-2-yl]-2,3-dihydro-1,4-benzodioxin-2-yl}methyl benzoate	G
38	12.66	C_26_H_30_O_13_	[M−H]^−^	549.1614	549.1603	2.026	255.0661, 153.0180, 135.0074, 119.0487	Isoliquiritin apioside	G
39	12.67	C_56_H_92_O_29_	[M−H]^−^	1227.5653	1227.5641	1.024	1065.51123, 933.47205、771.41693, 447.31555	3-*O*-β-d-xylopyranosyl(1 → 4)-[*β*-d-glucopyranosyl(1 → 2)]-*β*-d-glucopyranosyl-26-*O*-*β*-d-glucopyranosyl-(25*S*)-5*β*-furostane-22-methoxy-3*β*,26-diol	As
40	12.69	C_48_H_82_O_19_	[M−H]^−^	961.5383	961.5367	1.678	799.4863, 637.4363, 475.3780, 391.2874	20-Glc-Rf	P
41	12.75	C_45_H_74_O_19_	[M−H]^−^	917.4751	917.4741	1.138	755.4233, 593.3687, 553.3922, 364.0068, 319.1408	Timosaponin D	A
42	12.80	C_51_H_84_O_24_	[M−H]^−^	1079.5287	1079.5269	1.677	933.4645, 917.4766, 771.4186, 609.3615	Alliumoside B	A
43	12.87	C_23_H_24_O_10_	[M−H]^−^	459.1300	459.1286	3.107	255.0660, 153.0180, 135.0073, 119.0488	6'-acetylisoliquiritin	G
44	12.91	C_21_H_22_O_9_	[M−H]^−^	417.1190	417.1180	2.281	255.0661, 153.0179, 119.0487	Isoliquiritin	G
45	12.93	C_21_H_18_O_11_	[M−H]^−^	445.0776	445.0765	2.477	270.0490, 269.0455, 251.0349, 241.0509, 223.0393	Baicalin	S
46	12.95	C_26_H_30_O_13_	[M−H]^−^	549.1620	549.1603	3.137	255.0659, 153.0182, 135.0073, 119.0490	Licuraside	G
47	13.01	C_45_H_76_O_20_	[M−H]^−^	935.4857	935.4846	1.111	773.4354, 611.3803, 449.3252	Timosaponin E1	A
48	13.36	C_21_H_22_O_9_	[M−H]^−^	417.1195	417.1180	3.599	255.0662	Neoisoliquiritin	G
49	13.41	C_23_H_22_O_13_	[M−H]^−^	505.0992	505.0977	3.055	329.0667, 314.0435, 299.0198, 271.0250, 255.0291, 227.0344, 175.0237	5,6'-Dihydroxy-6,7-dimethoxyflavone 2'-*O*-*β*-d-glucuronide	S
50	13.53	C_21_H_20_O_11_	[M−H]-	447.0930	447.0922	1.861	271.0613, 243.0660	Dihydrobaicalin	S
51	13.60	C_42_H_72_O_14_	[M−H+HCOOH]^−^	845.4905	845.4893	1.381	799.4837, 637.4315, 475.3803, 273.3054	Ginsenoside Rg1	P
52	13.61	C_48_H_82_O_18_	[M−H+HCOOH]^−^	991.5499	991.5472	2.671	945.5428, 783.4907, 637.4326, 475.3786	Ginsenoside Re	P
53	13.69	C_35_H_36_O_15_	[M−H]^−^	695.1979	695.1970	1.199	549.1608, 531.1499, 255.0664, 153.0185, 135.0074, 119.0486	Licorice-glycoside B	G
54	13.81	C_36_H_38_O_16_	[M−H]^−^	725.2089	725.2076	1.722	549.1630, 531.1491, 255.0660, 153.0179, 135.0072, 119.0488	Licorice-glycoside A	G
55	13.92	C_21_H_18_O_11_	[M−H]^−^	445.0761	445.0765	− 0.961	270.0488, 269.0455, 249.0541, 241.0501, 225.0548	Apigenin 7-*O*-glucuronide	S
56	14.12	C_50_H_84_O_23_	[M−H]^−^	1051.5342	1051.5320	2.107	919.4982, 889.4860, 757.4376, 595.3851, 433.3344	Officinalisnin-II	As
57	14.20	C_45_H_76_O_19_	[M−H]^−^	919.4907	919.4897	1.103	757.4378, 595.3847, 433.3319	3-*O*-*β*-d-glucopyranosyl (1 → 2)-*β*-d-glucopyranosyl-26-*O*-*β*-d-glucopyranosyl-(25*S*)-5*β*-furostane-3*β*,22*α*,26-triol	As
58	14.24	C_50_H_84_O_23_	[M−H]^−^	1051.5327	1051.5320	0.709	919.4877, 889.4722, 757.4381, 594.6215, 418.5930	25-Epi-officinalisnin II	As
59	14.28	C_45_H_76_O_21_	[M−H]^−^	951.4787	951.4795	− 0.878	633.9669, 475.0884	(2*α*,3*β*,5*α*,22*S*)-26-(*β*-d-Glucopyranosyloxy)-2,5,22-trihydroxyfurostan-3-yl 4-*O*-*β*-d-glucopyranosyl-*β*-d-glucopyranoside	As
60	14.41	C_45_H_74_O_17_	[M−CO2−H]^−^	841.4950	841.4944	0.716	781.4773, 637.4346, 475.3789	Ginsenoside mRg1	P
61	14.45	C_48_H_74_O_20_	[M−H]^−^	969.4695	969.4690	0.525	922.5041, 825.9856, 760.4498, 471.1639, 351.0573	(3*β*,22*β*)-22-(*β*-d-Glucopyranosyloxy)-11-oxoolean-12-en-3-yl 2-*O*-*β*-d-glucopyranuronosyl-*β*-d-glucopyranosiduronic acid	G
62	14.46	C_56_H_92_O_27_	[M−H+HCOOH]^−^	1241.5817	1241.5797	1.584	1241.5817, 1195.5740, 1079.5382, 1033.5212, 917.4714, 755.4238, 455.1436	Ophiopojaponin G	O
63	14.55	C_21_H_18_O_11_	[M−H]^−^	445.0772	445.0765	2.409	270.0491, 269.0456, 251.0346, 241.0503, 225.0552, 223.0392	Isomer of baicalin	S
64	14.60	C_45_H_76_O_19_	[M−H]^−^	919.4914	919.4897	1.831	757.4382, 595.3838, 433.3329	3-*O*-*β*-d-Glucopyranosyl (1 → 2)-*β*-d-glucopyranosyl-26-*O*-*β*-d-glucopyranosyl-(25*R*)-5*β*-furostane-3*β*,22*α*,26-triol	As
65	14.61	C_51_H_86_O_24_	[M−H]^−^	1081.5433	1081.5425	0.740	919.4806, 757.4385, 595.3859	26-(Hexopyranosyloxy)-22-hydroxyfurostan-3-yl hexopyranosyl-(1 → 2)hexopyranosyl-(1 → 4)hexopyranoside	A
66	14.65	C_21_H_18_O_10_	[M−H]^−^	429.0821	429.0816	1.018	253.0505, 175.0236, 113.0229	Chrysin-7-*O*-*β*-d-glucuronid	S
67	14.79	C_22_H_20_O_11_	[M−H]^−^	459.0937	459.0922	3.338	283.0614, 269.0411, 268.0377, 241.0481, 175.0235	Oroxylin A-7-*O*-*β*-d-glucuronide	S
68	14.81	C_51_H_84_O_23_	[M−H]^−^	1063.5324	1063.5320	0.362	901.4807, 755.4263, 468.3537, 423.1946	Timosaponin BIV	A
69	14.91	C_22_H_20_O_12_	[M−H]^−^	475.0882	475.0871	2.247	299.0563, 284.0327	Isomer of hydroxylwogonin glucuronide	S
70	14.93	C_56_H_92_O_28_	[M−H]^−^	1211.5714	1211.5691	1.875	1079.5255, 917.4763, 865.0001, 755.4222	Timosaponin C1	A
71	15.02	C_45_H_76_O_19_	[M−H]^−^	919.4921	919.4897	2.571	841.4293, 757.4416, 595.3847, 459.0930	Timosaponin BII	A
72	15.59	C_22_H_20_O_11_	[M−H]^−^	459.0932	459.0922	2.205	283.0614, 269.0431, 268.0378, 240.0425, 175.0237	Wogonoside	S
73	15.70	C_57_H_94_O_27_	[M−H]^−^	1209.5912	1209.5899	1.088	1047.5446, 901.4795, 883.4755, 755.4213, 737.4127, 431.3182	(2*α*,3*β*,5*α*,25*R*)-2-Hydroxyspirostan-3-yl *β*-d-glucopyranosyl-(1 → 2)-[4-*O*-[(2*S*,3*R*,4*S*)-3-hydroxy-4-(hydroxymethyl)-4-methyltetrahydro-2-furanyl]-*β*-d-glucopyranosyl-(1 → 3)]-*β*-d-glucopyranosyl-(1 → 4)-*β*-d-galactopyranoside	A
74	15.91	C_51_H_84_O_22_	[M−H]^−^	1047.5380	1047.5371	0.868	901.4722, 885.4497, 755.4229	Protoneodioscin	As
75	16.06	C_51_H_84_O_22_	[M−H]^−^	1047.5382	1047.5371	1.107	901.4749, 883.4813, 755.4178, 413.2992	Protodioscin	As
76	16.09	C_50_H_84_O_25_	[M−H]^−^	1083.5170	1083.5218	− 4.452	1047.5375, 901.4825, 802.9248, 755.4275, 487.1885	(2α,3β,5α,22S,25R)-26-(β-d-Glucopyranosyloxy)-2,5,22-trihydroxyfurostan-3-yl β-d-xylopyranosyl-(1- > 3)-β-d-glucopyranosyl-(1- > 4)-β-d-galactopyranoside	As
77	16.29	C_50_H_84_O_22_	[M−H]^−^	1035.5374	1035.5371	0.289	903.5004, 889.4836, 757.4378, 595.3881, 433.3307	3-*O*-*α*-l-Rhamnopyranosyl(1 → 4)-[*β*-d-xylopyranosyl (1 → 2)]-*β*-d-glucopyranosyl-26-*O*-*β*-d-glucopyranosyl-(25*S*)-5*β*-furostane-3*β*,22*α*,26-trio	As
78	16.69	C_51_H_86_O_23_	[M−H]^−^	1065.5487	1065.5476	1.028	903.4990, 757.4362, 595.3870, 445.8120	(5*α*,22*R*)-26-(*β*-d-Glucopyranosyloxy)-22-hydroxyfurostan-3-yl 6-deoxy-*α*-l-mannopyranosyl-(1 → 4)-[*α*-l-mannopyranosyl-(1 → 2)]-*β*-d-glucopyranoside	A
79	16.87	C_48_H_72_O_22_	[M−H]^−^	999.4452	999.4431	2.041	837.3885, 351.0569	24-hydroxy-licoricesaponin A3	G
80	16.90	C_51_H_86_O_23_	[M−H]^−^	1065.5483	1065.5476	0.680	903.4954, 757.4395, 739.4266, 595.3826, 433.3332	3-*O*-*α*-l-rhamnopyranosyl (1 → 4)-[*β*-d-glucopyranosyl(1 → 2)]-*β*-d-glucopyranosyl-26-*O*-*β*-d-glucopyranosyl(25*R*)-5*β*-furostane-3*β*,22*α*,26-triol	As
81	17.24	C_45_H_76_O_18_	[M−H]^−^	903.4975	903.4948	0.363	757.4323, 595.3828, 433.3293	Aspacochioside A	As
82	17.47	C_45_H_76_O_18_	[M−H]^−^	903.4968	903.4948	2.256	757.4388, 595.3868, 433.3327	Isomer of aspacochioside A	As
83	17.53	C_44_H_64_O_19_	[M−H]^−^	895.3964	895.3958	0.619	456.4406, 429.6882, 351.0563	Hydroxy acetoxyglycyrrhizin	G
84	17.75	C_42_H_62_O_18_	[M−H]^−^	853.3855	853.3852	0.303	351.0568	22-Hydroxy-licoricesaponin G2	G
85	19.31	C_48_H_72_O_21_	[M−H]^−^	983.4494	983.4482	1.184	821.3983, 351.0575	Licorice-saponin A3	G
86	19.61	C_42_H_60_O_17_	[M−H]^−^	835.3760	835.3747	1.560	801.4187, 443.5862, 381.5747, 351.0575	formylglycyrrhizin acid	G
87	19.71	C_50_H_82_O_22_	[M−H]^−^	1033.5227	1033.5214	1.267	901.4716, 739.4283, 577.3704, 427.2860	3-*O*-*β*-d-xylopyranosyl (1 → 4)-[*β*-d-glucopyranosyl (1 → 2)]-*β*-d-glucopyranosyl-26-*O*-*β*-d-glucopyranosyl-(25*S*)-5*β*-furostane-20 (22)-ene-3*β*,26-diol	As
88	19.73	C_44_H_64_O_18_	[M−H]^−^	879.4027	879.4009	2.068	351.0570, 193.0346, 175.0236, 113.0229	22β-Acetoxyglycyrrhizin	G
89	19.78	C_45_H_74_O_18_	[M−H]^−^	901.4808	901.4791	1.873	739.4278, 577.3749, 356.5983	Xilingsaponin B	A
90	19.83	C_42_H_62_O_17_	[M−H]^−^	837.3911	837.3903	0.935	351.0570	Licorice-saponin P2	G
91	19.89	C_51_H_84_O_23_	[M−H]^−^	1063.5335	1063.5320	1.396	901.4749, 739.4255, 577.3785, 445.3186	3-*O*-*β*-d-xylopyranosyl(1 → 4)-[*β*-d-glucopyranosyl(1 → 2)-*β*-d-glucopyranosyl(1 → 2)]-*β*-d-glucopyranosyl-26-*O*-*β*-d-glucopyranosyl-(25*S*)-furostane-5-ene-22-methoxy-3*β*,26-diol	As
92	20.01	C_57_H_94_O_26_	[M-CO2-H]^−^	1149.6069	1149.6051	1.566	1149.6069, 1107.5963, 945.5444, 783.4910, 621.4361, 459.3843	Ginsenoside mRb1	P
93	20.04	C_47_H_80_O_17_	[M-H+HCOOH]^−^	965.4387	965.4377	1.025	919.4950, 758.4404, 497.1143, 435.1156	Gypenoside IX	P
94	20.09	C_48_H_76_O_19_	[M−H]^−^	955.4913	955.4897	1.699	793.4381, 731.4389, 613.3751, 569.3850, 523.3790, 455.3533	Ginsenoside Ro	P
95	20.11	C_56_H_92_O_25_	[M−CO2−H]^−^	1119.5964	1119.5946	1.675	1077.5857, 945.5567, 915.5332, 783.4905, 621.4422, 459.3855	Ginsenoside mRb2	P
96	20.13	C_45_H_74_O_17_	[M−H]^−^	885.4871	885.4842	3.199	739.4278, 577.3763, 484.2304	3-*O*-[{*α*-L-rhamnopyranosyl-(1 → 4)}{*β*-d-glucopyranosyl}]-26-*O*-[*β*-d-glucopyranosyl](25*S*)-5*β*-furost-20(22)-en-3*β*,26-diol	As
97	20.16	C_42_H_60_O_16_	[M−H]^−^	819.3818	819.3815	2.085	351.0568, 193.0346, 175.0237, 113.0229	Licorice-saponin E2	G
98	20.18	C_44_H_60_O_17_	[M−H]^−^	859.3739	859.3747	-0.904	837.3852, 797.3743, 351.0557	Methyllicorice-saponin Q2	G
99	20.19	C_42_H_62_O_17_	[M−H]^−^	837.3916	837.3903	1.521	351.0570	Macedonoside A	G
100	20.28	C_48_H_72_O_20_	[M−H]^−^	967.4548	967.4533	1.498	645.3641, 541.9834, 497.1151, 435.1159	(3*β*,22*β*)-23-Hydroxy-29-oxo-22,29-epoxyolean-12-en-3-yl 6-deoxy-*α*-*L*-mannopyranosyl-(1 → 2)-*β*-d-glucopyranuronosyl-(1 → 2)-*β*-d-glucopyranosiduronic acid	G
101	20.29	C_44_H_64_O_17_	[M−H]^−^	863.4081	863.4082	2.575	351.0566, 193.0343, 175.0241, 113.0229	22*β*-Acetoxyglycyrrhaldehyde	G
102	20.45	C_42_H_62_O_17_	[M−H]^−^	837.3915	837.3903	1.377	351.0568	Licorice-saponin Q2	G
103	20.47	C_39_H_66_O_14_	[M−H]^−^	757.4377	757.4369	1.132	595.3846, 465.2493, 357.2007, 271.7701, 161.0441	Anemarrhenasaponin I or II	A
104	20.50	C_42_H_62_O_16_	[M−H]^−^	821.3968	821.3954	1.678	352.0605, 351.0569, 193.0346, 175.0237, 113.0229	Glycyrrhizin acid	G
105	20.52	C_42_H_64_O_16_	[M−H]^−^	823.4128	823.4111	1.778	721.3478, 351.0557, 193.0344, 175.0237, 113.0226	Licorice-saponin J2	G
106	20.74	C_42_H_62_O_17_	[M−H]^−^	837.3918	837.3903	1.736	351.0573	Licorice-saponin G2	G
107	20.75	C_39_H_62_O_14_	[M−H+HCOOH]^−^	799.4072	799.4111	− 4.869	799.4072, 753.4070, 205.0709, 163.0600, 119.0335	Ophiopojaponin Ra	O
108	20.79	C_42_H_64_O_15_	[M−H]^−^	807.4177	807.4161	1.873	351.0572, 193.0346, 175.0236, 113.0230	Licorice-saponin B2	G
109	20.88	C_42_H_62_O_16_	[M−H]^−^	821.3971	821.3954	2.043	352.0600, 351.0571, 193.0346, 175.0237, 113.0229	Licorice-saponin H2	G
110	20.95	C_39_H_64_O_14_	[M−H]^−^	755.4227	755.4212	1.982	593.3716, 201.9089	Timosaponin AII	A
111	21.06	C_42_H_62_O_16_	[M−H]^−^	821.3972	821.3954	2.201	352.0616, 351.0570, 193.0346, 175.0238, 113.0229	Licorice-saponin K2	G
112	21.13	C_42_H_62_O_16_	[M−H]^−^	821.3970	821.3954	1.897	352.0634, 351.0573, 193.0345, 175.0237, 113.0229	Apioglycyrrhizin	G
113	21.57	C_42_H_62_O_15_	[M−H]^−^	805.4006	805.4005	0.090	351.0572, 193.0349, 175.0240, 113.0229	Licorice-saponin C2	G

A: Anemarrhe naerhizoma, S: Scutellariae radix, N: Nelumbinis folium, G: Glycyrrhizae radix, As: Asparagi radix, O: Ophiopogonis radix, P: Ginseng radix

Steroidal saponins in Erdong Decoction are partly from Asparagi radix and Anemarrhenae rhizoma, and partly from Ophiopogonis radix. But only two steroidal saponins from Ophiopogonis radix were tentatively identified by comparison with literature [11] (Table 1) and no saponins from Trichosanthis radix were identified in Erdong Decoction.

### Rapid identification of triterpenoid saponins

**Fig. 5:**
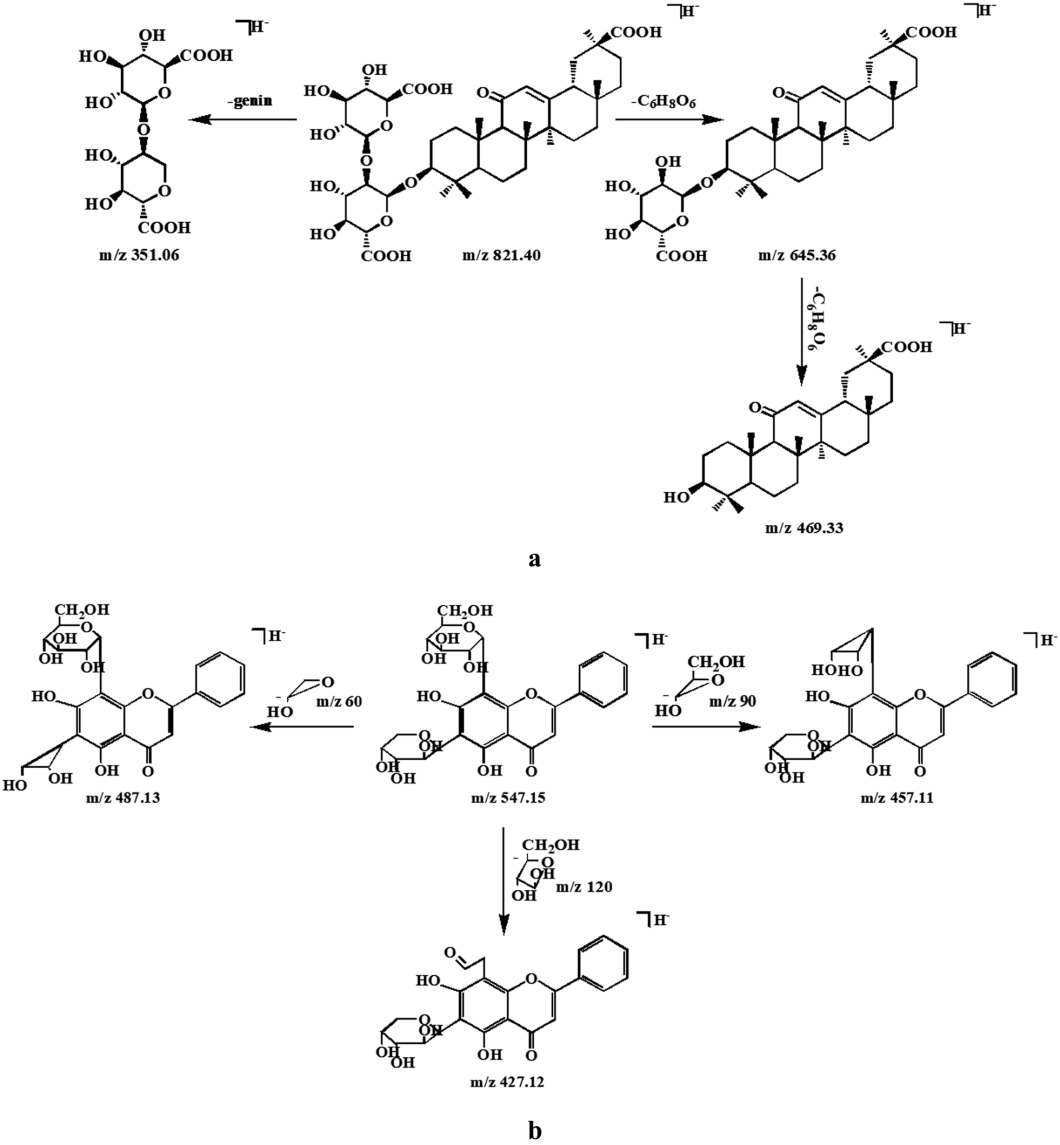
The proposed fragmentation pathways for **a** glycyrrhizin acid and **b** Chrysin 6-*C*-arabinoside-8-*C*-glucoside in negative mode

Triterpenoid saponins in Erdong decoction were derived from Glycyrrhizae radix and Ginseng radix. Glycyrrhizin acid as the mainly active compound in Glycyrrhizae radix [12], its MS/MS fragments mainly showed the fragment of disaccharides chain at *m/z* 351.057 and the weak signal of aglycone fragment at *m/z* 469.332. The fragmentation scheme of glycyrrhizin acid was further elaborated in Fig. 5a. In comparison to glycyrrhizin acid, its adjacent node of *m/z* 837.392 gave a MS/MS spectra of an identical disaccharides chain fragment, with different fragment of aglycone at *m/z* 485.330 (Fig. 4b). The node of *m/z* 837.392 was preliminarily deduced as glycyrrhizin acid analogue with one more hydroxyl group in the aglycone moiety of glycyrrhizin acid, finally annotated as macedonoside A by comparison with literature [12]. Based on the cluster, twenty-four triterpenoid saponins were rapidly tentative identification from Glycyrrhizae radix by comparison with literatures [12, 13], including 3 groups of isomers (Table 1), they were annotated in dark green in Fig. 2.

Ginsenosides could not be quickly identified by LC–MS/MS molecular networking under the condition of negative mode. Only 8 triterpenoid saponins from ginseng were tentatively identified by comparison with literatures [14, 15] (Table 1), they were annotated in purple in Fig. 2.

### Rapid identification of flavonoids

The flavonoids in Erdong decoction were derived from four herbs, Anemarrhenae rhizoma, Nelumbinis folium, Glycyrrhizae radix and Scutellariae radix. According to the difference of glycoside bond atoms, flavonoids in Erdong decoction were divided into two types. Identified flavonoids were annotated in blue for flavonoid *O*-glycosides and light blue for flavonoid *C*-glycosides (Fig. 2).

### Flavonoid *O*-glycosides

The flavonoid *O*-glycosides in the Erdong decoction are mainly from Scutellariae radix and Glycyrrhizae radix. The types of aglycone are mainly flavone and flavanone. It was well known that baicalin and wogonoside were mainly active components in Scutellariae radix [16, 17]. Peak 72 was identified as wogonoside by comparison with its standard compound, and its MS/MS spectra showed three characteristic fragments of *m/z* 283.061, *m/z* 268.038, and *m/z* 240.042, which in turn lost C_6_H_8_O_6_, CH_3_ and CO, the fragment of *m/z* 283.061 corresponding to the aglycone moiety of wogonoside by the loss of Da 176 (C_6_H_8_O_6_) from the [M−H]^−^ [18] (Additional file 1: Figure S3). The fragmentation scheme of wogonoside was further elaborated in Additional file 1: Figure S3. In comparison to wogonoside, its adjacent node of *m/z* 475.088 gave a MS/MS spectrum of different aglycone fragment at *m/z* 299.056 by the loss of Da 176 (C_6_H_8_O_6_), with one more hydroxyl group to the aglycone of wogonoside. The node of *m/z* 475.088 was annotated as the isomer of hydroxyl wogonoside according to literatures [16, 19] (Fig. 4c). Notably, another adjacent node of *m/z* 445.078 was connected to wogonoside in the molecular networking with a relatively low similarity (Fig. 4c). Comparing with wogonoside, the node of *m/z* 445.078 gave a MS/MS spectrum showing a different aglycone fragment at *m/z* 269.045 by the loss of Da 176 (C_6_H_8_O_6_), with one less methyl group to the aglycone of wogonoside. The node of *m/z* 445.078 was annotated as baicalin by comparison with standard compound. Basing on the cluster, forty-one flavonoid *O*-glycosides were tentatively identified from Scutellariae radix and Glycyrrhizae radix by comparison with literatures [12, 16, 17].

Some studies have shown that liquiritin and isoliquiritin are the active compounds in Glycyrrhizae radix [12]. It is noteworthy that some of isomers could not be distinguished by MS/MS and MN, but these isomers could be separated by retention time during LC–MS/MS analysis. Therefore, two groups of flavonoid isomers (peaks 9, 11, 44, 48, 14, 38, and 46) from Glycyrrhizae radix were tentatively identified by comparison with literatures [12, 13] (Table 1).

### Flavonoid *C*-glycosides

The flavonoid *C*-glycosides in Erdong decoction were mainly from Scutellariae radix and Anemarrhenae rhizoma. Taking peak 19 at *m/z* 547.146 as an example, at *m/z* 487.125, *m/z* 457.114, *m/z* 427.123 involved serial losses of 60 Da, 90 Da, 120 Da, revealed that these compounds were flavonoid *C*-glycosides with two attached saccharides: glucose and arabinose [16]. So peak 19 was identified as Chrysin 6-*C*-arabinoside-8-*C*-glucoside. The fragmentation scheme of Chrysin 6-*C*-arabinoside-8-*C*-glucoside was further elaborated in Fig. 5b and it shows special cleavage rule in the glucosyl part. In comparison to Chrysin 6-*C*-arabinoside-8-*C*-glucoside, its adjacent node of *m/z* 561.161 gave a MS/MS spectrum showing two characteristic fragments at *m/z* 471.130 and at *m/z* 441.118 by the loss of 90 Da, 120 Da, and so one more methyl group should be connected to the aglycone of Chrysin 6-*C*-arabinoside-8-*C*-glucoside. The node of *m/z* 561.161 was annotated as 5-hydroxy-7-methoxyflavone 6-*C*-arabinoside-8-*C*-glucoside or 7-hydroxy-5-methoxyflavone 6-*C*-arabinoside-8-*C*-glucoside [16] (Fig. 4d). Basing on the cluster, six flavonoid *C*-glycosides were tentatively identified from Scutellariae radix by comparison with literature [16].

Previous studies showed that the flavonoids from Anemarrhenae rhizoma were main xanthones, which was a special structure type of flavonoids, so it was not clustered with most of flavonoids in the molecular networking. Finally, 3 flavonoid *C*-glycosides were tentatively identified from Anemarrhenae rhizoma by comparison with literature [10] (Table 1).

### Identification of alkaloids

A total of 169 nodes were incorporated into the MS/MS molecular network (in the positive mode) of the Erdong decoction, rendering 15 molecular clusters and 88 unconnected nodes (Additional file 1: Figure S1). Besides the above three types of main compounds detected in Erdong decoction in negative mode, there are alkaloids from Nelumbinis folium mainly detected in positive mode. The mass spectrum of nuciferine at *m/z* 296.164 was detected and its MS/MS spectrum showed four characteristic fragments of *m/z* 265.123, *m/z* 250.098, *m/z* 234.103 and *m/z* 235.075 (Additional file 1: Figure S4). The fragmentation scheme of nuciferine was further elaborated in Additional file 1: Figure S4. It was well known that alkaloids were the major active compound of Nelumbinis folium [20], however, it was not shown in molecular networking and alkaloids could not be rapidly identified through the clusters in the LC–MS/MS molecular networking due to its various structural types. Finally, a total of 10 alkaloids were tentatively identified from Nelumbinis folium by comparison with literatures [20, 21] (Table 2).

**Table 2:** Tentative identification of the chemical constituents of Erdong decoction by UHPLC- MS in the positive mode

Peak no.	T_R_ (min)	Formula	Adduct ion	Experimental mass m/z	Theoretical mass m/z	Mass error (ppm)	Fragment ions	Identification	Source
114	6.43	C_19_H_23_NO_3_	[M+H]^+^	314.1746	314.1751	− 1.433	283.1324, 252.1144, 189.0908, 174.0670, 145.0645, 107.0494	Armepavine	N
115	6.89	C_18_H_21_NO_3_	[M+H]^+^	300.1591	300.1594	− 1.100	283.1324, 252.1143, 189.0909, 174.0671, 145.0647, 107.0494	NorarMepavine	N
116	9.06	C_18_H_19_NO_3_	[M+H]^+^	282.1485	282.1489	− 1.366	251.1062, 236.0828, 219.0801, 191.0853	*O*-Nornuciferine	N
117	10.84	C_38_H_44_N_2_O_6_	[M+H]^+^	625.3267	625.3272	− 0.885	566.4268, 489.2368, 325.0908, 206.1174, 163.0388, 121.0649	Dauricine	N
118	12.78	C_17_H_15_NO_2_	[M+H]^+^	266.1172	266.1176	− 1.485	249.0906, 219.0801, 191.0853	Anonaine	N
119	12.82	C_18_H_19_NO_2_	[M+H]^+^	282.1486	282.1489	− 0.834	265.1219, 250.0984, 234.1036	*N*-Methylnuciferine	N
120	12.96	C_18_H_17_NO_2_	[M+H]^+^	280.1330	280.1332	− 0.840	249.0907, 219.0803, 191.0854, 149.0233	Roemerine	N
121	13.07	C_19_H_21_NO_2_	[M+H]^+^	296.1643	296.1645	− 0.255	265.1218, 250.0984, 234.1035	Nuciferine	N
122	14.2	C_19_H_21_NO_3_	[M+H]^+^	312.1591	312.1594	− 0.300	265.1219, 250.0986, 234.1033	Pronuciferine	N
123	16.96	C_20_H_21_NO_4_	[M+H]^+^	340.1539	340.1543	− 1.278	269.1166, 233.1045, 215.0938, 197.0836, 178.0864	Tetrahydroberberine THB	N

N: Nelumbinis folium

## Discussion

In this study, the cluster of molecular networking in the negative mode (Fig. 2) was more obvious than that in the positive mode (Additional file 1: Figure S1). And more flavonoids, steroidal saponins, and triterpenoid saponins were tentatively identified in the negative mode than in positive mode. So, in this study, the flavonoids, steroidal saponins, and triterpenoid saponins in Table 1 were tentatively identified in the negative mode. The alkaloids were the major active compound of Nelumbinis folium, which were mainly detected in positive mode. And no cluster were observed in the molecular networking of the alkaloids, that might be due to the various types of structural framework of alkaloids, and it leads to the MS/MS fragments of alkaloids doesn’t have a certain similarity. Therefore, 10 alkaloids were tentatively identified in the positive mode from Nelumbinis folium by comparison with literatures.

According to the above results, LC–MS/MS molecular networking is suitable for the rapid identification of steroidal saponins, glycyrrhizin saponins, and flavonoids. Because of the stable structure of steroidal saponins and glycyrrhizin saponins, and special cleavage rule of flavonoid *C*-glycosides, their analogues in the LC–MS/MS molecular networking were obviously clustered with a high similarity. Based on the clusters, the structures of these compounds could be rapidly tentative identification by MN. In addition, the flavonoid *O*-glycosides obviously clustered in LC–MS/MS molecular networking, but the similarity between nodes was low, which might be due to different substituents sites on aglycones. Therefore, the identification of flavonoid *O*-glycosides could be facilitated by the combination of LC–MS/MS and molecular networking, but standard compounds are needed for the finally identification of isomers.

Notably, MS/MS-based molecular networking technique is not suitable for the rapid identification of compounds without cluster in MN. Steroidal saponins from Ophiopogonis radix and triterpenoid saponins from Ginseng radix in Erdong decoction couldn’t be rapidly identified, which might be due to their low content caused by both low formula ratio in Erdong decoction and low content in each herb itself. According to the unpublished quantification data by our laboratory, the content of saponins from Glycyrrhizae Radix Et Rhizoma, Anemarrhenae Rhizoma, Asparagi Radix are very high, whereas the content of saponins from Ophiopogonis Radix and Ginseng Radix Et Rhizoma are very low. The content of those compounds might be too low to generate fragment of aglycones in this study, so the MS/MS fragments of these compounds were not clustered in this study. The second type of compounds without cluster in the molecular networking is the alkaloids from Nelumbinis folium.

## Conclusions

In this study, the combination of LC-HRMS and molecular networking was applied to rapidly identify compounds in Erdong decoction as a case study to demonstrate the application of this technique in complex TCM formula. MS/MS-based molecular networking technique is very useful for the rapid identification of major components in CCF. Finally, 113 compounds were rapidly tentative identification in the negative mode by the MS/MS-based molecular networking, the types of these compounds mainly include steroidal saponin, triterpenoid saponins and flavonoids in Erdong decoction. MS/MS-based molecular networking greatly improves the efficiency of chemical components identification in CCF. In addition, 10 alkaloids were tentatively identified in the positive mode of Nelumbinis folium by comparison with literatures.

## Supplementary Information

**Additional file 1: Figure S1.** MS/MS molecular networking of Erdong decoction in the positive mode. **Figure S2.** The proposed fragmentation pathways and the MS/MS spectra for aspacochioside A in the negative mode. **Figure S3.** The proposed fragmentation pathways and the MS/MS spectra for wogonoside in the negative mode. **Figure S4.** The proposed fragmentation pathways and the MS/MS spectra for nuciferine in the positive mode.

## Data Availability

All data included in this article are available from the corresponding author upon request.

## Abbreviations

CCF: Chinese Classical Formula
UHPLC-LTQ-Orbitrap-MS/MS: Ultra-high pressure liquid chromatography-linear ion trap-orbitrap high resolution mass spectrometry
TCM: Traditional Chinese medicine
HRMS: High-resolution mass spectrometry
LC–MS: Liquid chromatography mass spectrometry
MN: Molecular networking
