# Supplementary material for: The combination of UHPLC-HRMS and molecular networking improving discovery efficiency of chemical components in Chinese Classical Formula
Source: Chin Med. 2021 Jul 2;16:50. doi: 10.1186/s13020-021-00459-6 (PMC8254261; doi:10.1186/s13020-021-00459-6)
Supplement: Supplementary file 1 — Additional file 1: Figure S1. MS/MS molecular networking of Erdong decoction in the positive mode. Figure S2. The proposed fragmentation pathways and the MS/MS spectra for aspacochioside A in the negative mode. Figure S3. The proposed fragmentation pathways and the MS/MS spectra for wogonoside in the negative mode. Figure S4. The proposed fragmentation pathways and the MS/MS spectra for nuciferine in the positive mode. [file 13020_2021_459_MOESM1_ESM.docx]

The combination of UHPLC-HRMS and molecular networking improving discovery efficiency of chemical components in Chinese Classical Formula

Xiaoxia Xue ^1,2,#^, Qishu Jiao ^1,#^, Runa Jin ^1^, Xueyuan Wang ^1,3^, Pengyue Li ^1^, Shougang Shi ^4^, Zhengjun Huang ^4^, Yuntao Dai ^1,2,^*, and Shilin Chen ^1^

**Author information**

^1^ Institute of Chinese Materia Medica, China Academy of Chinese Medical Sciences, 100700, Beijing, China; [xxxue1003@163.com](mailto:xxxue1003@163.com) (XX); [qs_jiao1108@bucm.edu.cn](mailto:qs_jiao1108@bucm.edu.cn) (QJ); [jrn11330@163.com](mailto:jrn11330@163.com) (RJ); [774740773@qq.com](mailto:774740773@qq.com) (XW); [pyli@icmm.ac.cn](mailto:pyli@icmm.ac.cn) (PL); slchen@icmm.ac.cn (SC)

^2^ Shanxi University of Chinese Medicine, Jinzhong, 030619, Shanxi, China

^3^ Modern Research Center for Traditional Chinese Medicine, Shanxi University, Taiyuan, 030006, Shanxi, China

^4^ Sunflower Pharmaceutical Group (Xiangyang) Longzhong Co.Ltd, Xiangyang, 441003, Hubei, China; 497869216@qq.com (SS); xfhzj2007@163.com (ZH)

^#^ Co-first author

^*^ Correspondence: [ytdai@icmm.ac.cn](mailto:ytdai@icmm.ac.cn) (YD)


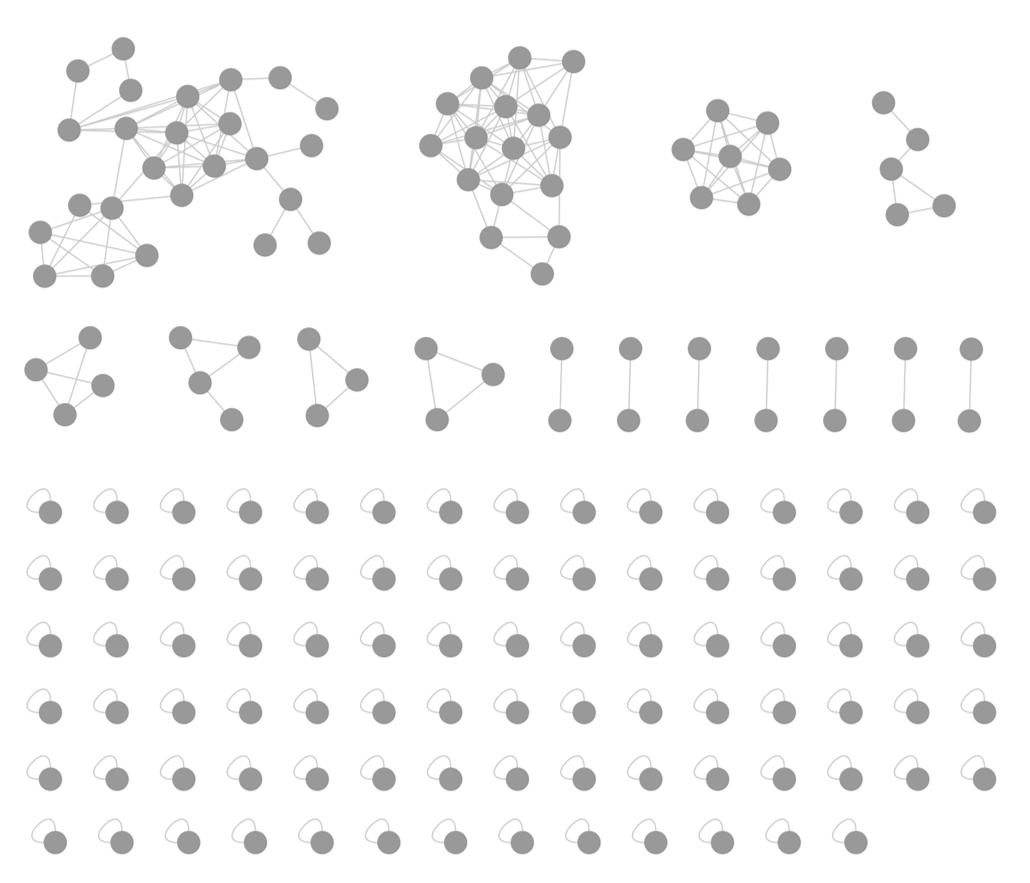


**Figure S1.** MS/MS molecular networking of Erdong decoction in the positive mode.


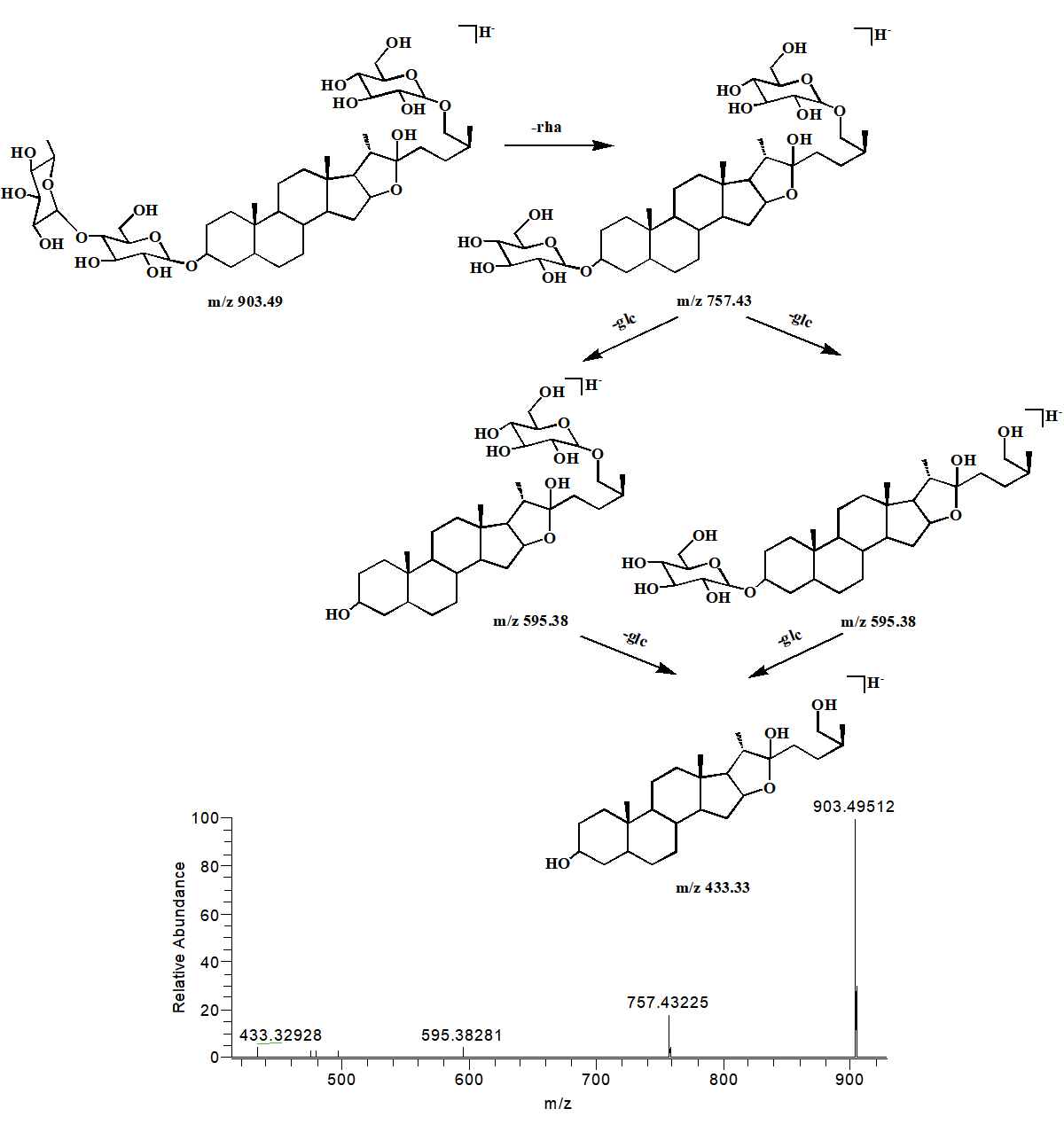


**Figure S2.** The proposed fragmentation pathways and the MS/MS spectra for aspacochioside A in the negative mode.


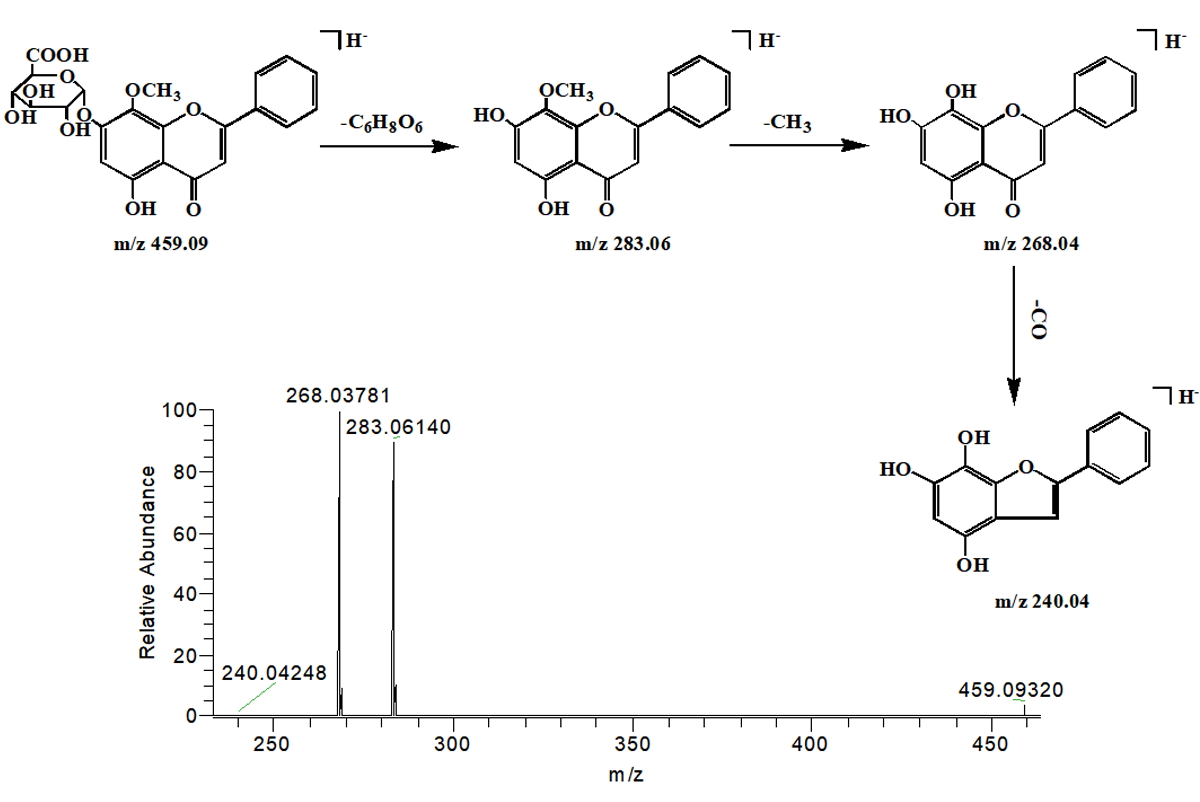


**Figure S3.** The proposed fragmentation pathways and the MS/MS spectra for wogonoside in the negative mode.


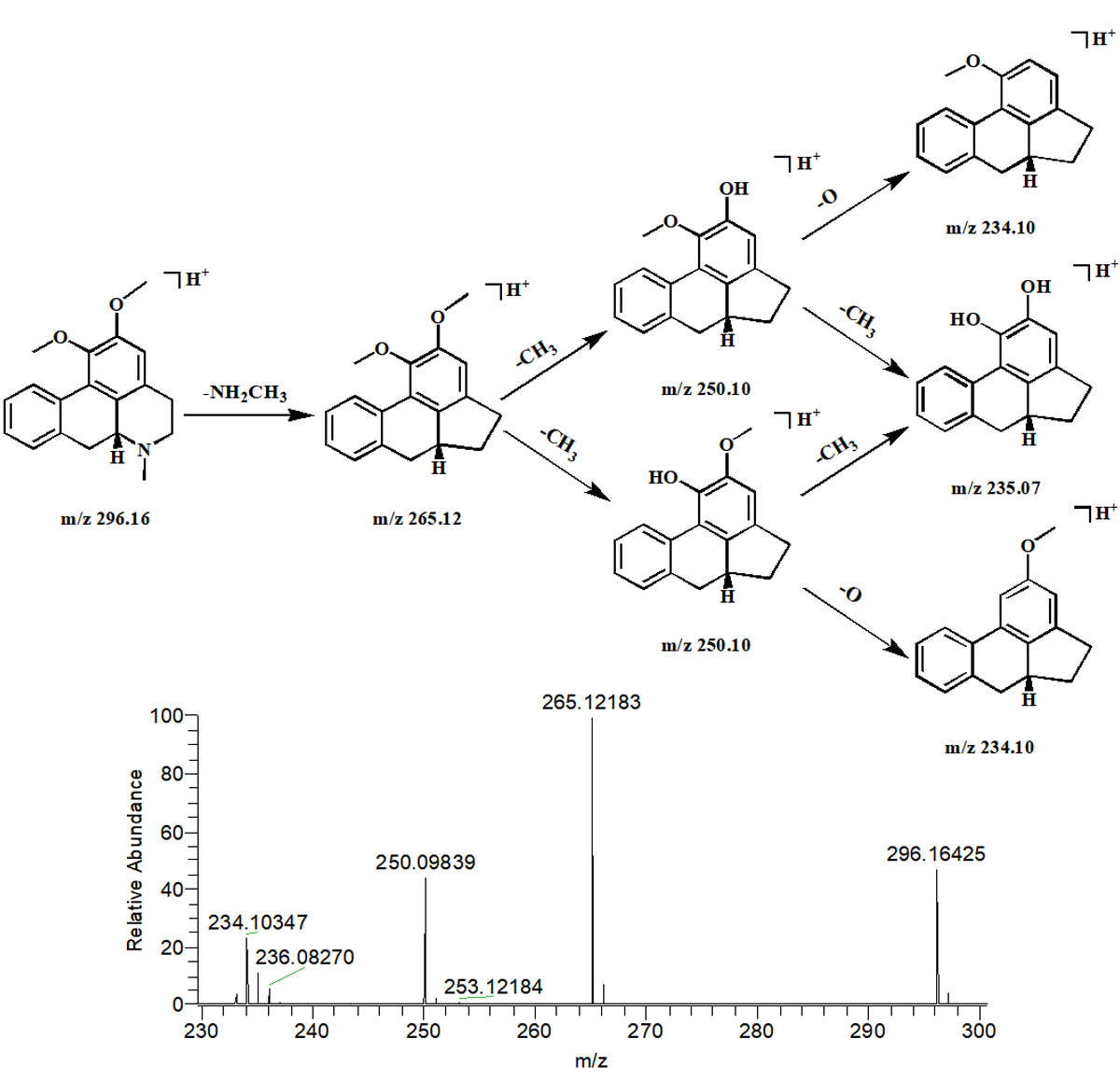


**Figure S4.** The proposed fragmentation pathways and the MS/MS spectra for nuciferine in the positive mode.
